# Long non-coding RNA ZFAS1 interacts with miR-150-5p to regulate Sp1 expression and ovarian cancer cell malignancy

**DOI:** 10.18632/oncotarget.14663

**Published:** 2017-01-14

**Authors:** Bairong Xia, Yan Hou, Hong Chen, Shanshan Yang, Tianbo Liu, Mei Lin, Ge Lou

**Affiliations:** ^1^ Department of Gynecology, the Affiliated Tumor Hospital, Harbin Medical University, Harbin, China; ^2^ Department of Biostatistics, Public Health School, Harbin Medical University, Harbin, China

**Keywords:** ovarian cancer, long non-coding RNA, ZFAS1, miR-150-5p, Sp1

## Abstract

We reported that long non-coding RNA ZFAS1 was upregulated in epithelial ovarian cancer tissues, and was negatively correlated to the overall survival rate of patients with epithelial ovarian cancer in this study. While depletion of ZFAS1 inhibited proliferation, migration, and development of chemoresistance, overexpression of ZFAS1 exhibited an even higher proliferation rate, migration activity, and chemoresistance in epithelial ovarian cancer cell lines. We further found miR-150-5p was a potential target of ZFAS1, which was downregulated in epithelial ovarian cancer tissue. MiR-150-5p subsequently inhibited expression of transcription factor Sp1, as evidence by luciferase assays. Inhibition of miR-150-5p rescued the suppressed proliferation and migration induced by depletion of ZFAS1 in epithelial ovarian cancer cells, at least in part. Taken together, our findings revealed a critical role of ZFAS1/miR-150-5p/Sp1 axis in promoting proliferation rate, migration activity, and development of chemoresistance in epithelial ovarian cancer. And ZFAS1/miR-150-5p may serve as novel markers and therapeutic targets of epithelial ovarian cancer.

## INTRODUCTION

Epithelial ovarian cancer (EOC) is most common type of ovarian cancer. Ovarian cancer has the highest mortality rate of all gynecologic malignancy, accounting for 5–6% of cancer-related death in women. Approximately, 21,550 women develop ovarian cancer annually in the United States [1]. Although cytoreductive surgery followed by a combination of platinum- and taxane-based chemotherapy has been widely used as the standard treatment of advanced ovarian cancer, most cases developed chemoresistance, and eventually died from this disease [2]. Because the molecular pathology of ovarian cancer remains unclear, survival rates of patients largely depend on the stage of ovarian cancer at diagnosis. Thus, a better understanding on molecular pathology of ovarian cancer will facilitate development of early detection approaches and novel therapies for patients with ovarian cancer.

Both genetic and epigenetic alterations, contribute to initiation and progression of human cancer. Over the few past years, accumulating evidence has shown epigenetic regulations, such as non-coding RNAs, histone modification, DNA modification, and recently identified RNA modification play an essential role in both physiological and pathological processes [3–6]. Long non-coding RNAs (lncRNAs) are a diverse class of RNAs with more than 200 nucleotides in length that do not encode proteins [7]. Genome-wide transcriptional analysis has revealed the high prevalence of lncRNAs in all the transcripts [8]. Regarding to the functions, lncRNA has been found to be an important epigenetic modulator with sensory, guiding, and scaffolding capacities [9, 10]. Thus, lncRNAs have gained increasing attention as a critical regulator of normal physiology and disease development, such as stem cell differentiation and tumorigenesis [11, 12]. On the other hand, lncRNAs expression was dysregulated in many diseases across different tissue types [11, 13, 14]. LncRNAs can be detected in various human body fluids, such as serum, plasma, and saliva, suggesting lncRNAs may serve as new biomarkers for disease diagnosis and prognosis without invasion procedures [15–17].

LncRNA zinc finger antisense 1 (ZFAS1) is a transcript antisense to the 5′ end of the gene Znfx1. Most recently, dysregulation of ZFAS1 have been observed in patients with acute myocardial infarction and various human cancers, including breast cancer, colorectal cancer, gastric cancer and hepatocellular carcinoma [18–22]. For instance, ZFAS1 was expressed at a high level in normal mammary gland and was downregulated in breast tumors. siRNA-mediated depletion of ZFAS1 in mammary epithelial cells increased cellular proliferation and promoted differentiation, indicating a crucial role of ZFAS1 in mammary development and breast cancer progression [18]. In contrast, ZFAS1 was also reported to be significantly upregulated in colorectal cancer compared to paired normal tissues. By interacting with CDK1/cyclin B1 complex and promoting destabilization of p53, ZFAS1 enhanced proliferation and inhibited apoptosis of colorectal cancer cells [20]. These findings highlight the role of ZFAS1 in regulation of physiological and pathological processes with high tissue specificity, but whether ZFAS1 is involved in the ovarian cancer development remains largely unknown.

In this study, we measured the expression levels of ZFAS1 in 66 EOC tissues and 10 normal epithelial ovarian tissue samples, and found that ZFAS1 was upregulated in EOC tissues and was correlated to poor prognosis. Via siRNA-mediated knockdown and overexpression, we demonstrated ZFAS1 promoted cell proliferation, migration, invasion, and development of chemoresistance to Cisplatin and Paclitaxel in EOC cell lines. To investigate the potential targets of ZFAS1, we used DIANA TOOLS and found the miR-150-5p was directly regulated by ZFAS1, which further promoted ovarian cancer progression by regulating Sp1 [23].

## RESULTS

### ZFAS1 is upregulated in EOC tissues and cell lines, and associated with poor prognosis

**Figure 1:**
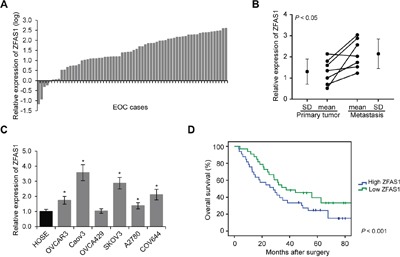
ZFAS1 is upregulated in EOC and associated with poor prognosis **A**. ZFAS1 expression levels was determined by qRT-PCR in 66 EOC tissues and 10 normal epithelial tissues samples (*p*<0.001). **B**. Relative expression levels of ZFAS1 in seven paired primary and metastatic EOC tissues by qRT-PCR (*p*<0.05). **C**. Relative expression levels of ZFAS1 in 6 EOC cell lines (OVCAR3, Caov3, OVCA429, SKOV3, A2780, and COV644), as compared to those in human ovarian surface epithelial (HOSE cells, *p<0.05). **D**. Kaplan-Meier survival curves in high and low ZFAS1 expression groups (*p*<0.001).

In order to assess the role of ZFAS1 in EOC oncogenesis, we first analyzed ZFAS1 expression levels in 66 EOC tissue specimens by qRT-PCR. The value was normalized to 10 normal ovarian epithelial tissues as shown in Figure 1A, we found that the expression levels of ZFAS1 were significantly higher in EOC tissues (*p*<0.001). Among these clinical EOC patients, six cases were diagnosed with peritoneal metastases. We then analyzed ZFAS1 expression in paired primary and metastatic EOC tissues. The expression levels of ZFAS1 were significantly upregulated in metastatic specimens (Figure 1B, *p*<0.05). In addition, we determined the levels of ZFAS1 in six EOC cell lines (OVCAR3, Caov3, OVCA429, SKOV3, A2780, and COV644). The expression of ZFAS1was significantly higher in all six cell lines, compared to those in human ovarian surface epithelial (HOSE) cells (Figure 1C). Among the six cell lines, Caov3 and SKOV3 cells had the highest ZFAS1expression. We analyzed the clinical relevance of ZFAS1 in EOC patient survival, as shown in Figure 1D, the EOC patients with high ZFAS1 had poor prognosis.

### ZFAS1 enhances EOC cell proliferation, migration, invasion and chemoresistance

**Figure 2:**
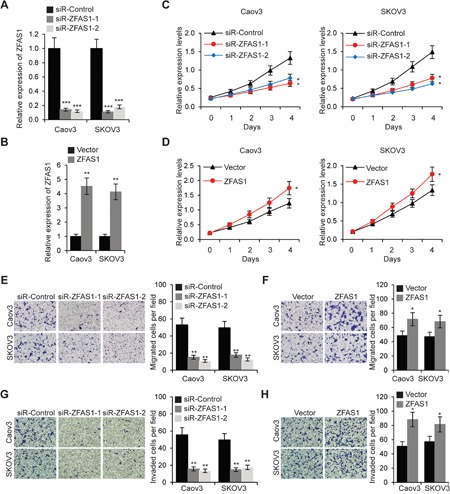
ZFAS1 promotes EOC cell proliferation, migration and invasion **A**. Relative expression levels of ZFAS1 was determined by qRT-PCR in Caov3 and SKOV3 cells transfected with ZFAS1siRNA (siR-ZFAS1-1 and siR-ZFAS1) or control siRNA (siR-Control). **B**. Relative expression levels of ZFAS1 was determined by qRT-PCR inCaov3 and SKOV3 cells transfected with ZFAS1 overexpression plasmids or empty vector. **C**. MTT assays of Caov3 and SKOV3 cells transfected with ZFAS1siRNA or siR-Control. **D**. Caov3 and SKOV3 cells were transfected with ZFAS1 or vector for 48 h, then MTT assays were performed. **E**. Migration assays in Caov3 and SKOV3 cells transfected with ZFAS1siRNA or control siRNA. **F**. Caov3 and SKOV3 cells were transfected with ZFAS1 or vector, then cell migration assays were performed. **G**. Invasion assays in Caov3 and SKOV3 cells transfected with ZFAS1siRNA or control siRNA. **H**. Caov3 and SKOV3 cells were transfected with ZFAS1 or vector, then cell invasion assays were performed.*p<0.05, **p<0.01 and ***p<0.001 compared with control cells.

Next, we sought to determine whether ZFAS1 could affect the proliferation, migration and invasion of EOC cells. ZFAS1 expression was downregulated and upregulated by ZFAS1 siRNA and plasmid transfection, respectively. The levels of ZFAS1 were determined by qRT-PCR (Figure 2A and 2B). We found that decrease of ZFAS1 significantly reduced growth rate, and increase of ZFAS1 promoted cell proliferation as determined by MTT assays in both Caov3 and SKOV3 cell lines (Figure 2C and 2D). Additionally, migration and invasion assays demonstrated that downregulation of ZFAS1 significantly inhibited EOC cell migration and invasion abilities, while upregulation of ZFAS1 enhanced cell metastasis (Figure 2E to 2H).

**Figure 3:**
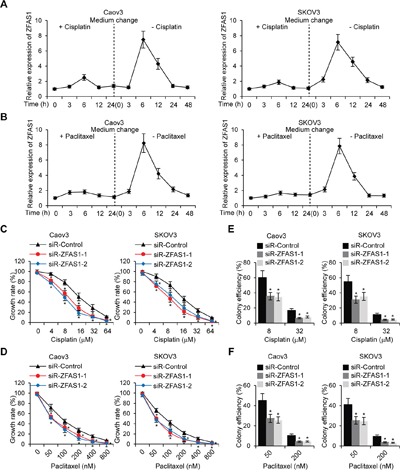
Knockdown of ZFAS1 reduces drug-resistance in EOC cells **A, B**. Caov3 and SKOV3 cells werecultured in complete medium with Cisplatin (8 μM) or Paclitaxel (50 nM)for 24 h, then medium was changed without Cisplatin and Paclitaxelfor 48 h. The ZFAS1 expression were examined by qRT-PCR at different time points indicated. **C, D**. MTT cell growth rate were performed in ZFAS1 knockdown Caov3 and SKOV3 cells treated with different concentration of Cisplatin orPaclitaxelindicated. **E, F**. Caov3 and SKOV3 cells were transfected with ZFAS1 siRNA or control siRNA for 48 h, then colony formation assays were performed with treatment of Cisplatin or Paclitaxel indicated.*p<0.05 compared with control cells.

To investigate the effect of ZFAS1 on EOC cell chemoresistance, we first treated wild type Caov3 and SKOV3cells with 8 μM Cisplatin or 50 nM Paclitaxel for 24 h, then changed to growing medium and cultured cells without Cisplatin and Paclitaxel for another 24 h. ZFAS1 expression were examined at different time points. The results revealed that ZFAS1 was significantly upregulated after 6 h of treatment (Figure 3A and 3B). We next treated EOC cells with different concentrations of Cisplatin and Paclitaxel, and performed MTT assays to examine cell growth rate. As shown in Figure 3C and 3D, knockdown of ZFAS1 resulted in a higher sensitivity to Cisplatin or Paclitaxel treatment in EOC cells. Consistently, colony formation assays demonstrated that knockdown of ZFAS1 significantly inhibited EOC cell colony forming efficiency with Cisplatin or Paclitaxel treatment (Figure 3E and 3F).

### MiR-150-5p is a target of ZFAS1

**Figure 4:**
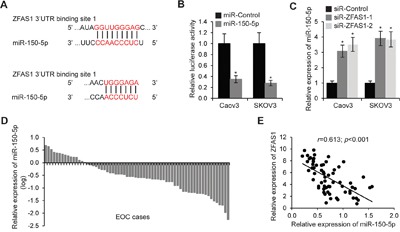
MiR-150-5p is a target of ZFAS1 **A**. The putative binding sites of ZFAS1 and miR-150-5p. **B**. The luciferase reporter plasmid containing ZFAS1 was co-transfected with miR-150-5p or miR-Control in Caov3 and SKOV3 cells. Luciferase activities were determined at 48 h after transfection. The relative luciferase activities normalized to *Renilla* activity.*p<0.05 compared with control cells. **C**. Relative expression of miR-150-5p in Caov3 and SKOV3 cells transfected with ZFAS1 siRNA. *p<0.05 compared with control cells. **D**. Expression levels of miR-150-5p in 66 EOC tissues relative to 10 normal epithelial tissues samples (*p*<0.001). **E**. The correlation of the expression levels of ZFAS1 and miR-150-5p (*r*=0.613; *p*<0.001).

We used DIANA TOOLS LncBase to predict potential ZFAS1 targets. Multiple miRNAs were found, we selected miR-150-5p for further analysis since miR-150-5p has previously been reported to play a pivotal role in a series of cancer types [24–27]. MiR-150-5p was predicted binding to two potential sites in ZFAS1 (Figure 4A). We inserted ZFAS1 with miR-150-5p predicted binding sites into luciferase reporter vectors and co-transfected with miR-150-5p or miR-Control into Caov3 and SKOV3 cells. As compared with control, the luciferase activity was significantly inhibited when miR-150-5p transfection (Figure 4B). In ZFAS1 knockdown EOC cells, miR-150-5p expression was found upregulated (Figure 4C). We further analyzed miR-150-5p expression in 66 EOC samples by normalizing 10 normal ovarian epithelial tissues, as shown in Figure 4D, expression levels of miR-150-5p was decreased in most of EOC patient specimens. Furthermore, spearman correlation analysis revealed a reverse correlation between ZFAS1 and miR-150-5p expression (Figure 4E). Taken together, these results indicate that miR-150-5p is a target of ZFAS1.

### MiR-150-5p inhibits EOC cell proliferation, migration, invasion and chemoresistance

**Figure 5:**
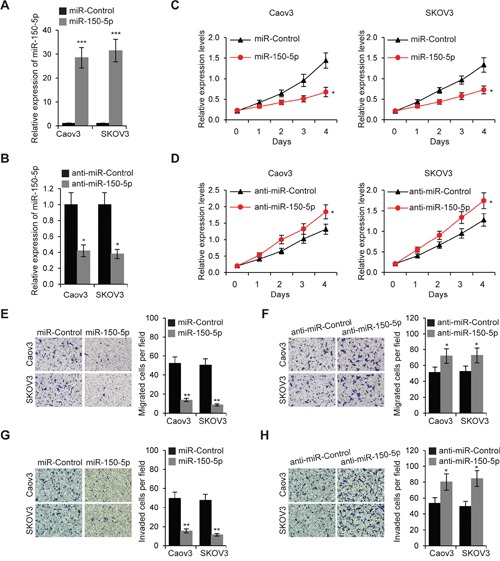
MiR-150-5p inhibits EOC cell proliferation, migration and invasion **A**. Relative expression levels of miR-150-5p were determined by qRT-PCR in Caov3 and SKOV3 cells transfected with miR-150-5porcontrol miRNA (miR-Control). **B**. Relative expression levels of miR-150-5p was determined by qRT-PCR inCaov3 and SKOV3 cells transfected with miR-150-5p inhibitor (anti-miR-150-5p) or control miRNA inhibitor (anti-miR-Control). **C**. MTT assays of Caov3 and SKOV3 cells transfected with miR-150-5p or miR-Control. **D**. Caov3 and SKOV3 cells were transfected with anti-miR-150-5p or anti-miR-Control for 48 h, then MTT assays were performed. **E**. Migration assays in Caov3 and SKOV3 cells transfected with miR-150-5porcontrol miRNA. **F**. Caov3 and SKOV3 cells were transfected with anti-miR-150-5p or anti-miR-Control, then cell migration assays were performed. **G**. Invasion assays in Caov3 and SKOV3 cells transfected with miR-150-5porcontrol miRNA. **H**. Caov3 and SKOV3 cells were transfected with anti-miR-150-5p or anti-miR-Control, then cell invasion assays were performed. *p<0.05, **p<0.01 and ***p<0.001 compared with control cells.

To explore the effect of miR-150-5p on EOC cell proliferation, we increased or decreased miR-150-5p expression in Caov3 and SKOV3 cells by miR-150-5p or anti-miR-150-5p transfection, respectively (Figure 5A and 5B). We then performed MTT assays and found that miR-150-5p inhibited cell proliferation, and miR-150-5p inhibitor enhanced EOC cell proliferation (Figure 5C and 5D). Furthermore, we investigated whether miR-150-5p regulates Caov3 and SKOV3 cell migration and invasion. As shown in Figure 5E to 5H, miR-150-5p decreased and anti-miR-150-5p increased the number of migrated cells. Consistently, upregulation of miR-150-5p reduced and miR-150-5p inhibitor promoted EOC cell invasion ability (Figure 5G and 5H).

**Figure 6:**
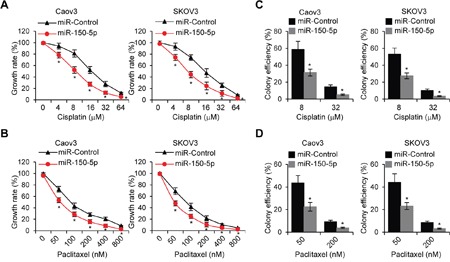
MiR-150-5p reduces chemoresistance in EOC cells **A, B**. MTT cell growth rate were performed in miR-150-5p transfectedCaov3 and SKOV3 cells treated with different concentration of Cisplatin or Paclitaxel indicated. **C, D**. Caov3 and SKOV3 cells were transfected with miR-150-5p or control miRNA for 48 h, then colony formation assays were performed with treatment of Cisplatin or Paclitaxel indicated. *p<0.05 compared with control cells.

Next, we studied whether miR-150-5p affects EOC cell chemoresistance. Caov3 and SKOV3 cells were transfected with miR-150-5p or miR-Control for 48 h. These cells were then subjected to MTT assays after treatment of different concentration of Cisplatin and Paclitaxel. The results revealed that overexpression of miR-150-5p leaded to a higher sensitivity to Cisplatin or Paclitaxel in EOC cells (Figure 6A and 6B). Moreover, colony formation assays demonstrated that miR-150-5p significantly inhibited EOC cell colony forming efficiency with Cisplatin or Paclitaxel treatment (Figure 6C and 6D).

### MiR-150-5p directly targets transcription factor *Sp1* (Specificity protein 1)

**Figure 7:**
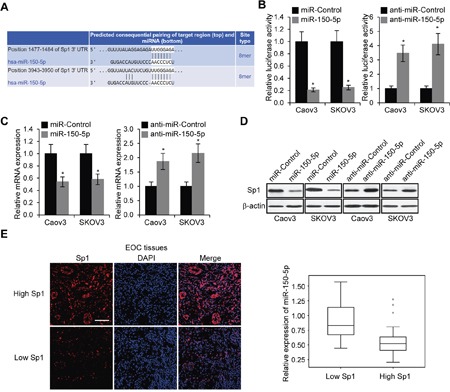
MiR-150-5p directly targets Sp1 **A**. The putative binding sites of miR-150-5p in the Sp1 3′-UTR regions. **B**. Luciferase activities in Caov3 and SKOV3 cells 48 h after co-transfected with Sp1 3′-UTRluciferase reporter plasmids and miR-150-5p, miR-Control, or their inhibitors, respectively. *p<0.05 compared to control cells. **C**. mRNA expression levels of Sp1 in Caov3 and SKOV3 transfected miR-150-5p, miR-Control, anti-miR-150-5p or anti-miR-Control, respectively. *p<0.05 compared to control cells. **D**. Levels of Sp1 protein was determined by Western blot in Caov3 and SKOV3 cells transfected with miR-150-5p, miR-Control, anti-miR-150-5p or anti-miR-Control, respectively. **E**. Immunofluorescent staining of Sp1 in EOC tissues (Scale bar, 50 μm).MiR-150-5p expression in low and high Sp1 expression groups (*p*<0.001).

We employed the TargetScan to predict the potential target genes of miR-150-5p, and found that *Sp1* was one of the functionally relevant target genes [28]. Besides TargetScan, we used DIANA TOOLS and microRNA.org to confirm that Sp1 was predicted as a potential target of miR-150-5p. There were two miR-150-5p binding sites in Sp1 3′-UTR (Figure 7A). In order to further confirm that Sp1 was a direct target gene of miR-150-5p, we constructed luciferase reporter plasmid with the Sp1 3′-UTR region. The luciferase reporter plasmid was co-transfected with miR-150-5p or anti-miR-150-5p, and luciferase activity was examined. MiR-150-5p significantly inhibited and anti-miR-150-5p elevated luciferase activity (Figure 7B). We next examined the mRNA and protein levels of Sp1 in Caov3 and SKOV3 cells transfected with miR-150-5p or its inhibitor. The results revealed that miR-150-5p remarkably reduced and anti-miR-150-5p boosted both Sp1 mRNA and protein expression levels in both EOC cell lines (Figure 7C and 7D). Furthermore, we performed immunofluorescent staining for Sp1 expression and examined the miR-150-5p levels in low and high Sp1 groups, and found that miR-150-5p was downregulated in high Sp1 group (*p*<0.001). Collectively, these results suggested that miR-150-5p inhibited the expression of Sp1 by targeting its 3′-UTR region.

### ZFAS1 promotes EOC cells through miR-150-5p mediated Sp1

**Figure 8:**
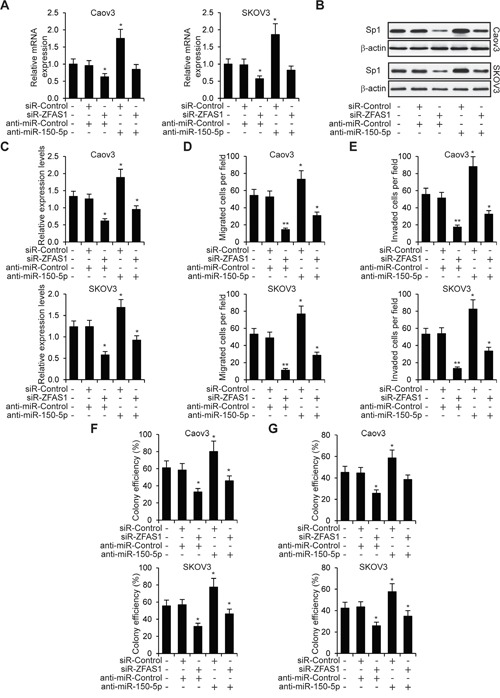
ZFAS1 promotes EOC cells through miR-150-5p mediated Sp1 **A, B**. Relative mRNA and protein expression in five group of Caov3 and SKOV3 cells (no transfection, siR-Control + anti-miR-Control, siR-ZFAS1 + anti-miR-Control, siR-Control + anti-miR-150-5p and siR-ZFAS1 + anti-miR-150-5p) determined by qRT-PCR and western blot. **C, D, E**. MTT cell proliferation, migration and invasion assays in five group of Caov3 and SKOV3 cells (no transfection, siR-Control + anti-miR-Control, siR-ZFAS1 + anti-miR-Control, siR-Control + anti-miR-150-5p and siR-ZFAS1 + anti-miR-150-5p). **F, G**. Colony formation assays in Caov3 and SKOV3 cells treated with Cisplatin (8 μM) or Paclitaxel (50 nM).*p<0.05 and**p<0.01 compared to no transfection cells.

The above results showed that ZFAS1 promoted and miR-150-5p inhibited EOC malignancy and chemoresistance. ZFAS1 directly targeted miR-150-5p and Sp1 was a miR-150-5p target gene. It is possible that ZFAS1/miR-150-5p/Sp1axis exerted its functions on EOC cells. To test this hypothesis, we first knocked down ZFAS1 in Caov3 and SKOV3 cells, and examined Sp1 expression. As we expected, both Sp1 mRNA and protein expression was downregulated due to upregulation of miR-150-5p in ZFAS1 downregulated cells. We then inhibited miR-150-5p expression in ZFAS1 knockdown EOC cells by anti-miR-150-5p transfection (Figure 8A and 8B). We found Sp1 expression was partially rescued in both Caov3 and SKOV3 cells (Figure 8A and 8B). We next transfected anti-miR-150-5p into ZFAS1 knockdown Caov3 and SKOV3 cells, and tested cell proliferation, migration and invasion abilities. As shown in Figure 8C to 8E, knockdown of ZFAS1 significantly decreased cell proliferation, migration and invasion. MiR-150-5p inhibition partially rescued EOC cell malignancy. Furthermore, we treated EOC cells with Cisplatin (8 μM) or Paclitaxel (50 nM) when ZFAS1 knockdown. Consistently, knockdown of ZFAS1 promoted Caov3 and SKOV3 cell chemosensitivity. Further inhibition of miR-150-5p abolished chemosensitivity induced by downregulation of ZFAS1 (Figure 8F and 8G). These findings demonstrated that ZFAS1 promotes EOC cell malignancy and chemoresistance, at least in part, via miR-150-5p mediated Sp1.

## DISCUSSION

ZFAS1 is recently identified lncRNA. Previously, ZFAS1 was reported to be upregulated in colorectal cancer and hepatocellular carcinoma, but to be upregulated in breast cancer [18, 20, 21]. In this study, we found ZFAS1 was highly expressed in EOC tissues, which further highlighted the distinct roles of ZFAS1 in different tissues. Notably, ZFAS1 was found extremely stable with a half-life of more than 16 h in mammary epithelial cell, and exhibited no evidence of degradation at 16 h post transcriptional inhibition [18].

We found miR-150-5p was a target of ZFAS1. MiR-150-5p, located on chromosome 19q13, was shown to be downregulated in hepatocellular carcinoma tissues, and may suppress the progression of hepatocellular carcinoma by inhibition of the migration of hepatoma cell by targeting MMP14 (matrix metallopeptidase 14) [26, 27]. MiR-150 has also been reported to be involved in development of several human cancers, such as cervical cancer, breast cancer, colorectal cancer and leukemia [25, 29–31]. In the present study, we found miR-150-5p was a target of ZFAS1 by using DIANA TOOLs. In contrast to ZFAS1, miR-150-5p was downregulated in EOC tissues compared to non-cancerous tissues. Furthermore, miR-150-5p significantly inhibited the proliferation and migration of EOC cells. Giving that lncRNAs are capable to bind to the target DNA, RNA, and protein, whether ZFAS1 directly binds to miR-150-5p or the precursor miR-150 remains to be investigated. Of note, knockdown of miR-150-5p did not fully rescue the inhibited proliferation and migration activity of EOCs caused by depletion of ZFAS1, indicating there might be other targets of ZFAS1 that were involved in this process.

In addition, we found ZFAS1 was required for the chemoresistance of EOC cells, while miR-150-5p made the EOC cells more sensitive to Cisplatin and Paclitaxel. We further demonstrated that miR-150-5p directly targeted transcription factor Sp1 by bioinformatics tools and luciferase assays [28]. EOC tissues with high levels of miR-150-5p showed significantly lower expression of Sp1. As previously reported, Sp1 was crucial for the transcription initiation of CLDN4 (claudin-4) and chemoresistance [32–34]. However, Sp1 alone was not sufficient to promote CLDN4 expression, and ovarian cancer cells that expressed high level of CLDN4 exhibited high histone H3 acetylation and low DNA methylation at CLDN4 promoter region, indicating epigenetic regulations were involved in the development of chemoresistance in ovarian cancer cells [32].

In conclusion, we found that ZFAS1 was overexpressed in EOC tissues, and ZFAS1/miR-150-5p/Sp1 axis promoted the proliferation, migration, invasion and development of chemoresistance in EOC cells. Therefore, ZFAS1 and miR-150-5p may represent novel therapeutic targets and diagnostic biomarkers for EOC.

## MATERIALS AND METHODS

### Human samples

66 EOC tissue specimens were collected from patients who underwent surgery at the Department of Obstetrics and Gynecology of Harbin Medical University Cancer Hospital between 2012 and 2014. 10 normal epithelial ovarian tissue sections were from cervical cancer patients over 45 years old in the same period. The pathological results routinely diagnosed by three pathologist. None of the patients were treated with chemotherapy or radiotherapy before operation. The histopathological diagnoses were performed according to the World Health Organization criteria. The percentage of tumor cells in all primary EOC samples are more than 50%. All fresh specimens were stored at −80°C for further use. This study was approved by the Medical Ethics Committee of Harbin Medical University Cancer Hospital and all patients provided informed consent. Clinical characterization of all EOC patients were analyzed in Table 1.

**Table 1:** Association of ZFAS1 and clinical feature of 60 EOC patients

Characteristics	Total	ZFAS1 expression		*P* value
		Low	High	
Age (years)				0.195
>55	32	13	19	
≤55	28	17	11	
Stage				0.018
I+II	26	18	8	
III+IV	34	12	22	
Grade				0.060
1	22	7	15	
2+3	38	23	15	
Tumor size				0.047
>1	42	17	25	
≤1	18	13	5	
Histology				0.295
Serous	35	15	20	
Non-serous	25	15	10	
Lymph node				0.002
Negative	40	26	14	
Positive	20	4	16	
MiR-150-5p expression				0.000
High	30	25	5	
Low	30	5	25	
Sp1 expression				0.000
High	30	7	23	
Low	30	23	7	

### Cancer cell lines and primary normal epithelial cells

The human EOC cell lines (OVCAR3, Caov3, OVCA429, SKOV3, A2780, and COV644) and normal Human Ovarian Surface Epithelial (HOSE) cells were supplied by China Center for Type Culture Collection (CCTCC). EOC cells were cultured in Dulbecco’s modified Eagle’s medium (DMEM; Gibco-BRL, Gaithersburg, MD) supplemented with 10% fetal bovine serum (FBS) and antibiotics (Gibco-BRL). HOSE cells were cultured in medium containing 1:1 mixture of MCDB 105 and M199 medium (Sigma, St. Louis, MO). All cells were incubated at 37°C under a humidified atmosphere containing 5% CO_2_.

### Quantitative real-time PCR (qRT-PCR)

Total RNA was extracted using Trizol reagent (Invitrogen, Carlsbad, CA) according to the manufacturer’s instructions. qRT-PCR analyses for *ZFAS1* and *Sp1* were performed by using QIAGEN OneStep RT-PCR kits (Qiagen, Valencia, CA) and SYBR Green real-time PCR. The mRNA level of *β-actin* was measured as an internal control. To quantitate miR-150-5p expression, total RNA was polyadenylated and reverse transcribed using TaqMan MicroRNA Reverse Transcription Kit and TaqMan miRNA assays (Applied Biosystems, Foster City, CA). U6 small nuclear RNA was used as the internal control. Relative expression of the tested genes was calculated and normalized using the 2^−ΔΔCt^ method. Primers were as follows: *ZFAS1* forward, 5′ AAGCCACGTGCAGACATCTA 3′, reverse, 5′ CTACTTCCAACACCCGCATT 3′; *Sp1* forward, 5′ TCATACTGTGGGAAACGCTT 3′, reverse 5′ GACACTCAGGGCAGGCAAA 3′; *β-actin* forward, 5′ TGACGGGGTCACCCACACTGTGCCCATCTA3′, reverse, 5′ CTAGAAGCATTTGCGGTGGACGATGGAGGG 3′.

### Transfection and luciferase assays

All oligonucleotides were transfected into EOC cells at a final concentration of 50 nM using HiPerFect transfection reagent according to the product manual (Qiagen). The full-length ZFAS1 and 3′UTR of Sp1 gene containing the putative miR-150-5p biding sites was amplified by PCR and was inserted into the psiCHECK2 vector (Promega, Madison, WI, USA). The coding sequences of ZFAS1 were generated by PCR and cloned into pCDNA3.1 (+) vector (Invitrogen) to generate pCDNA3.1- ZFAS1 plasmids. The plasmids were all transfected using Lipofectamine LTX according to the manufacturer's instructions.

Cells were seeded in triplicate in 24-well plates one day before transfection for the luciferase assays. 48 h after transfection, the cells were harvested and lysed, and the luciferase activity assayed using the dual-luciferase assay kit (Promega). Normalized luciferase activity was reported as luciferase activity/*Renilla* luciferase activity.

### 3-(4,5-Dimethylthiazol-2-yl)-2,5-diphenyl tetrazolium bromide (MTT) assays

At 48 h after transfection or treatment, the cells were seeded into 96-well plates at 2000 per well in a final volume of 100 μl. Then at 0, 1, 2, 3 and 4 days, 25 μl of MTT (Promega) stock solution was added to each well and incubated for 4 h. The absorbance was measured at 570 nm.

### Transwell migration and invasion assays

In vitro cell migration and invasion assays were performed using 24-well Transwell chambers (8-μm pores, BD Biosciences, San Jose, CA). The transfected EOC cells (5 × 10^4^ cells per well) were cultured in the top chamber with 100 μl 1% FBS medium. 500 μl complete media with 10 % FBS was added into the lower chamber. After 24 h of culture, the medium from the chamber and the Transwell was removed, and the chamber was gently wiped with a cotton swab. The migrated cells were fixed in 4 % paraformaldehyde, stained with crystal violet solution and counted under a microscope in six fields. The procedure for the cell invasion assays was similar to the cell migration assays, except that the Transwell membranes were precoated with Matrigel (BD Biosciences).

### Colony formation assays

The transfected EOC cells were seeded in 6-well plates (300 cells per well) overnight, then treated with different concentration of Cisplatin or Paclitaxel for 1 h and incubated in complete medium without Cisplatin and Paclitaxel for ten days. The cells were then washed with PBS, fixed with 10% formalin, and stained with 0.5% crystal violet (Sigma). The assays were repeated in five replicates. The colony efficiency was calculated as following: colony efficiency = (clone number/total cell number)/(control clone number/control total cell number) × 100%.

### Western blot

Western blot was performed as described previously [33]. Briefly, Total protein was extracted by RIPA buffer (50 mM Tris–HCl pH 7.4,150 mM NaCl, 1% NP-40, 1% sodium deoxycholic acid, 0.1% SDS, 1 mM phenylmethylsulfonyl fluoride, protease inhibitor cocktail; Santa Cruz Biotechnology). The total extracts were separated using 10% SDS-polyacrylamide gels and electrophoretically transferred to polyvinylidene difluoride membranes (PVDF, Bio-Rad, Hercules, CA). The membranes were probed with a primary antibody against human Sp1 or β-actin from Santa Cruz, followed by horseradish peroxidase (HRP) -conjugated secondary antibody (Santa Cruz). Bound antibody was detected using the Supersignal West Pico ECL chemiluminescence kit (Thermo scientific, Rockford, IL).

### Tissue immunofluorescent staining

EOC tissues were fixed in 4% formaldehyde, embedded in paraffin wax, and then cut into 5 μm sections. Samples were de-paraffinized in xylene and rehydrated. After blocking endogenous peroxidase and performing antigen retrieval, tissue slides were blocked in 1% BSA and incubated with antibodies against Sp1 (Santa Cruz) overnight at 4°C, followed by goat anti-rabbit IgG conjugated to Texas Red (Santa Cruz). DAPI was used for nuclear staining.

### Statistical analysis

Statistical analyses were performed by SPSS Windows version 19. Data was expressed as mean ± SD of the experiments performed in triplicate. One-Way ANOVA followed by Tukey’s post hoc was performed to determine the significance of difference among groups. Differences were considered significant at *p<0.05, **p<0.01 and ***p<0.001.
